# Epidemiology of the first-ever cardiovascular event in people with type 1 diabetes: a retrospective cohort population-based study in Catalonia

**DOI:** 10.1186/s12933-023-01917-1

**Published:** 2023-07-14

**Authors:** Gabriel Giménez-Pérez, Clara Viñals, Manel Mata-Cases, Bogdan Vlacho, Jordi Real, Josep Franch-Nadal, Emilio Ortega, Dídac Mauricio

**Affiliations:** 1 Section of Endocrinology, Department of Medicine, Hospital General de Granollers, Granollers, Spain; 2 School of Medicine and Health Sciences, Universitat Internacional de Catalunya, Sant Cugat del Vallès, Spain; 3 Department of Endocrinology & Nutrition, Hospital Clínic de Barcelona, Barcelona, Spain; 4 DAP-Cat Group, Unitat de Suport a La Recerca Barcelona Ciutat, Institut Universitari d’Investigació en Atenció Primària Jordi Gol, Barcelona, Spain; 5 CIBER of Diabetes and Associated Metabolic Diseases (CIBERDEM), Instituto de Salud Carlos III (ISCIII), Barcelona, Spain; 6 Primary Health Care Center La Mina, Gerència d’Àmbit d’Atenció Primària Barcelona Ciutat, Institut Català de La Salut, Sant Adrià de Besòs, Spain; 7 Primary Health Care Center Raval Sud, Gerència d’Àmbit d’Atenció Primària Barcelona Ciutat, Institut Català de La Salut, Barcelona, Spain; 8 CIBER of Physiopathology of Obesity and Nutrition, ISCIII, Madrid, Spain; 9 Department of Endocrinology & Nutrition, Hospital de la Santa Creu i Sant Pau, Barcelona, Spain; 10 Department of Medicine, University of Vic - Central University of Catalonia, Vic, Spain

**Keywords:** Type-1 diabetes; cardiovascular disease, Peripheral vascular disease, Ischemic heart disease, Cerebrovascular disease, Heart failure

## Abstract

**Background:**

Knowledge of the characteristics of first-ever cardiovascular events in type 1 diabetes may impact primary prevention strategies. This study describes the first-ever manifestation of cardiovascular disease (CVD) in patients with type 1 diabetes (T1D) in Catalonia (Spain) and evaluates differences according to age and sex.

**Methods:**

Retrospective cohort study of patients with T1D > 30 years without CVD before 2010 registered in the SIDIAP database. The occurrence of a first cardiovascular event up to the end of 2016, the type of CV event and associations with baseline characteristics were analysed.

**Results:**

Of 8412 patients, 884 suffered a first CV event (incidence rate 1.62 per 100 persons-years). Overall, peripheral vascular disease (39.5%) was the most frequent event. We observed a higher proportion of heart failure in women (21.7%) than in men (10.1%). In women, heart failure was the most frequent event in those > 65 years (40.5%). Decreased glomerular filtration rate (hazard ratio [HR] 5.42 [95% CI 4.32;6.80]), elevated albumin/creatinine ratio (HR 3.39 [95% CI [2.47;4.66], microvascular complications (HR 3.27 [95% CI 2.85;3.75]), and hypertension (HR 3.21 [95% CI [2.80;3.67]) were most strongly associated with a first CV event. HbA1c > 7.0% was associated with incident CVD only in patients aged < 55/60 years.

**Conclusions:**

Peripheral artery disease in the whole cohort, and heart failure in elder subjects are the most frequent first-ever CVD events in T1D in our region. These findings deserve to be taken into account when considering primary prevention measures and when estimating CV risk in people with T1D.

**Supplementary Information:**

The online version contains supplementary material available at 10.1186/s12933-023-01917-1.

## Background

Cardiovascular disease (CVD) is the main cause of morbidity and mortality among people with type 1 diabetes (T1D), even in those with good metabolic control [1, 2]. Despite relevant progress in diabetes care and increased life expectancy, it is estimated that people with T1D experience CV events about 10 years earlier and have on average 2 to fourfold increased risk compared with the general population [2–4]. Even with a decrease in all-cause and CVD mortality in recent decades, those who are younger (< 40 years) do not appear to experience the same benefits as the older population [5].

Duration of T1D has been considered a risk factor for CVD and mortality; furthermore, those who develop T1D before 10 years of age are at higher risk of CVD, especially females [6, 7]. Moreover, women with T1D have a 40% greater excess risk of all-cause mortality and twice the excess risk of fatal and nonfatal CV events compared with men [8].

No single risk factor has been solely implicated in the increased CVD risk in T1D. However, it is known that there is a strong association between increasing numbers of CV risk factors not at target levels and an increased risk of poor CV outcomes in patients with T1D [9]. Age and glycaemic control are the most important risk factors for CVD, with HbA1c being the strongest modifiable risk factor [10]. Furthermore, the different manifestations of CVD may be associated with different risk factors, with evidence suggesting that HbA1c may be the strongest risk factor for CVD death, congestive heart failure and angina, while age may be the strongest risk factor for acute myocardial infarction (MI), silent MI and coronary revascularisation [11].

Recently, our group described the clinical status, the control of CV risk factors and CV protective treatments in more than 15,000 patients with T1D in Catalonia (Spain). Overall, we found that there was room for improvement in glycaemic and lipid control in a significant number of T1D patients with CVD [12]. However, data regarding the incidence of CVD, including peripheral artery disease and heart failure, in a Mediterranean population with T1D is lacking.

Thus, our objective was to describe the first clinical manifestation of CVD and to evaluate the differences by age and sex in a large population-based cohort of patients with T1D in Catalonia (Spain), and to estimate the incidence of different manifestations of CVD in this population.

## Methods

This was a retrospective cohort study of patients with T1D registered in the SIDIAP (Information System for the Development of Primary Care Research) database [13]. SIDIAP is a primary healthcare database which captures pseudo-anonymised information of approximately 5.8 million people in Catalonia registered with a family physician from the *Institut Català de la Salut* (ICS, Catalan Institute of Health). ICS is the main provider of primary healthcare services in the Catalan Health System (CatSalut), managing 286 primary care teams, representing 74% of the total population. Although patients with T1D usually attend specialist care settings bound to a hospital facility, prescriptions for chronic treatments and glucose control materials are provided by the primary care centres, guaranteeing that regardless of where clinical care is provided, the records of patients with T1D of any age in the ICS database can be considered as complete. The SIDIAP database includes data from the primary care electronic medical records (demographics, diagnoses, clinical variables, prescriptions, referrals, and laboratory results). It also includes medications dispensed in pharmacy offices, and incorporates data of hospital discharges obtained from the Basic Minimum Set of Data (BMSD). The SIDIAP database has previously been used to conduct multiple observational studies that evaluate clinical characteristics and outcomes in type 1 [12, 14] and type 2 [15, 16] diabetes in Catalonia.

### Study population

The study population consisted of subjects older than 30 years with a registered diagnosis of T1D (International Classification of Diseases 10 [ICD-10] code E10) without a registered diagnosis of CVD (Additional file 1: Table S1) or atrial fibrillation (I48.*) prior to January 1, 2010. To refine the selection of patients with T1D, we excluded those patients treated with glucose-lowering agents other than insulin and patients with a concomitant diagnosis of other types of diabetes (E11, E13, E14) if the age of diagnosis was above 30 years. The outcome of a first fatal or non-fatal CV event was defined as the presence of a first registered diagnosis of CVD from January 1, 2010 to December 31, 2016. The type and date of this first CVD diagnosis was recorded. To describe the type of CV event, diagnostic codes were grouped in coronary, cerebrovascular, peripheral vascular and heart failure events, as specified in Additional file 1: Table S1. In addition, the date of death of persons who died during the study period was recorded. Unfortunately, specific causes of death were not available in this study.

### Clinical and laboratory data

If not otherwise stated, the last registered clinical and laboratory data during the year 2009 were used. Data collected for the present analysis included age, sex, duration of diabetes (2010 minus year of diabetes diagnosis), smoking habit (current, former or never), body mass index (BMI), HbA1c, blood pressure, blood lipids, estimated glomerular filtration rate (eGFR) using the CKD-EPI (Chronic Kidney Disease Epidemiology Collaboration) formula, the urinary albumin/creatinine ratio, the presence of registered microvascular complications (E10.2 to E10.4, N08.3, E36.0 or G63.2), treatment with statins, anti-hypertensive drugs or aspirin, and the type of insulin used. The deprivation level according to the MEDEA (socioeconomic status) index [17] was also collected in quintiles with the primary care centre as a unit of analysis and with the first quintile as the least deprived population. Low HDL was defined as < 40 mg/dl for men and < 50 mg/dl for women, while atherogenic dyslipidaemia was defined as the presence of low HDL cholesterol and triglycerides ≥ 150 mg/dl [18].

Patients were categorised in age group according to their age on January 1, 2010 as follows: young (Y): < 35 years; early adulthood (EA): 35 to 55/60 (men/women) years; middle adulthood (MA): 55/60 to 65 (men/women); young old (YO): 66 to 75 years and middle-to-very old (MVO): > 75 years. This age grouping is a pragmatic and clinical approach that considers the age range at which systematic cardiovascular risk evaluation is recommended (35–75 years) in our Health Care System, the age limit to define premature events according to sex, i.e., < 55 years for men and < 60 years for women [19]**,** and a common clinical recommendation to individualise CV prevention in younger (under 40 years) and older (above 75 years) individuals.

To analyse the relationship between HbA1c and the incidence of CV events, HbA1c levels were categorised as follows: < 7.0%, 7.0–8.0%, and > 8.0%.

As a retrospective study using pseudo-anonymous routinely collected health data, informed consent was not obtained from participants according to Spanish regulations on observational studies. The study was approved by the Ethics Committee of the Primary Healthcare University Research Institute (IDIAP) Jordi Gol (Barcelona, Spain), approval number P17/087 on 15/03/2017.

### Statistical analysis

The baseline characteristics were described as frequencies and percentages for categorical variables, while for continuous variables the mean and standard deviation (SD), or median and interquartile ranges (IQR) were calculated. The cumulative incidence and incidence rates of CV manifestations were computed using the exact method. For each event of interest, the overall incidence rate and age-sex specific incidence was computed as the number of incident events divided by the persons-years (PY) during the follow-up and was expressed as per 100 PY. Hazard ratios (HR) and 95% confidence intervals (95% CI) were computed for each baseline characteristic. In the comparison between groups (sex), p-value was calculated using the Fisher exact test for qualitative variables and the independent samples t-test for quantitative variables. In the comparison between age groups, the p-trend was calculated. The *compare Group*s R Package (Version 4.6.0) [20] was used to perform group descriptions for several variables and to estimate each HR. All statistical analyses were performed using the free R statistical software, version 3.6.1 (www.jstatsoft.org/v57/i12/).

## Results

The final sample consisted of 8,412 patients (42.3% women) with a median age of 42.3 years (IQR 35.7–51.8) and a mean duration of diabetes of 10.9 ± 10.0 years. The clinical and laboratory parameters of the entire population and according to the pre-specified age categories are shown in Additional file 1: Table S2. As shown, mean HbA1c for the whole population was 7.87% (SD ± 1.64), and 28.7% of patients had an HbA1c level < 7%. The median values of CV risk factors were within an acceptable range for primary prevention, except for smoking with a prevalence of 38.7%, which was higher at younger ages. Most patients were in their early adulthood (35–55 years; 62.2%). Sex ratios differed between age groups with a higher proportion of women at older ages. Systolic and diastolic blood pressure showed a significant increase across age-groups. In contrast, most lipid parameters did not differ between age-groups and no clinically relevant differences were observed in HbA1c levels. Microalbuminuria prevalence, kidney dysfunction (filtration rate < 60 ml/min/1.73 m^2^), and the proportion of microvascular complications worsened with increasing age. The prescription of CV prevention drugs (statins, anti-hypertensive and anti-platelet drugs) was higher in older age groups. On the other hand, prescription of long-acting insulin analogs and short-acting insulins was higher in younger age groups.

### Incidence rates and type of first non-fatal cardiovascular event

During the study period, 557 men (11.5%) and 327 (9.2%) women suffered a first CV event during 54,422 person-years (PY) (incidence rate 1.62 per 100PY, cumulative incidence 10.51%). Table 1 shows the incidence rate and cumulative incidence of a first CV event according to sex and age groups. As can be observed, cumulative incidence increased steadily along age groups in both sexes. Additionally, from the initial 8412 (4853 men and 3559 women) individuals at risk at baseline, 297 men (6.1%) and 193 (5.4%) women died (Additional file 1: Table S5). Before the date of death, 186 (37.9%) of these individuals (similar percentage in men and women, 37.7% and 38.3%, respectively) had had a cardiovascular event (Additional file 1: Table S6). Unfortunately, the specific cause of death was not available in this study.

**Table 1:** Incidence rate and cumulative incidence of the first-ever cardiovascular event according to sex and age groups

	At risk (n)	Events (n)	Person-time (persons-years)	Incidence rate (per 100 persons-years)	Cumulative incidence (%)
All	8412	884	54,422	1.62	10.51
Sex					
Women	3559	327	23,233	1.41	9.18
Men	4853	557	31,188	1.79	11.48
Age groups					
Y < 35 years	1810	36	12,495	0.29	1.99
EA 35–55/60 years	5231	479	34,503	1.39	9.16
MA 55/60–65 years	696	148	4108	3.60	21.26
YO 65–75 years	411	123	2226	5.53	29.93
MVO > 75 years	264	98	1090	8.99	37.12
Age and sex groups					
Y-Women	737	15	5096	0.29	2.04
Y-Men	1073	21	7400	0.28	1.96
EA-Women	2283	176	15,182	1.16	7.71
EA-Men	2948	303	19,321	1.57	10.23
MA-Women	161	25	1029	2.43	15.53
MA-Men	535	123	3078	4.00	22.99
YO-Women	211	53	1199	4.42	25.12
YO-Men	200	70	1027	6.82	35.0
MVO-Women	167	58	727	7.98	34.73
MVO-Men	97	40	363	11.02	41.24

Age groups: *Y* young, *EA* early adulthood, *MA* middle adulthood, *YO* young old, and *MVO* middle-to-very old

**Fig. 1:**
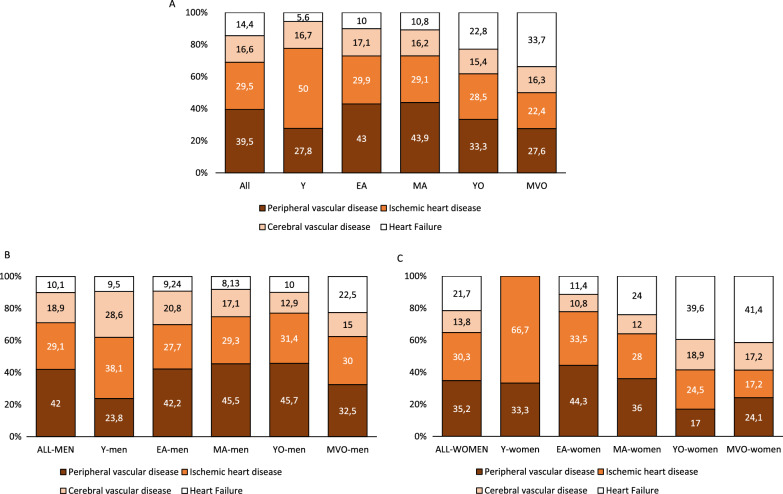
Frequency distribution of the different types of first cardiovascular event according to sex and age groups in patients with type 1 diabetes. **A** All population; **B** men; **C** women

Figure 1 shows the frequency distribution of the different manifestations of the first CV event according to sex and age groups. As shown, the most frequent type of CV event in the entire population was peripheral vascular disease (349 events; 39.5%), followed by ischemic heart disease (261 events; 29.5%). The occurrence of cerebrovascular disease (147 events; 16.6%) and heart failure were similar (127 events; 14.4%). While the proportion of ischemic heart disease as a first CV manifestation was similar between male and female patients, the distribution of all the other events differed by sex, particularly due to a higher proportion of heart failure in women (71 events, 21.7% of all events in women) than in men (56 events, 10.1% of all events in men). The type of the first CV event also differed according to age groups. In men, the proportion of the different types of events remained rather stable across age groups, except for young (< 35 years) men who showed a higher proportion of ischemic heart and cerebrovascular disease (Fig. 1b). In women, however, there was a decrease in the proportion of ischemic heart and peripheral vascular disease with increasing age and an increase of cerebrovascular disease and, specially, heart failure (Fig. 1c).

### Baseline characteristics in association with cardiovascular events stratified by age and sex

**Fig. 2:**
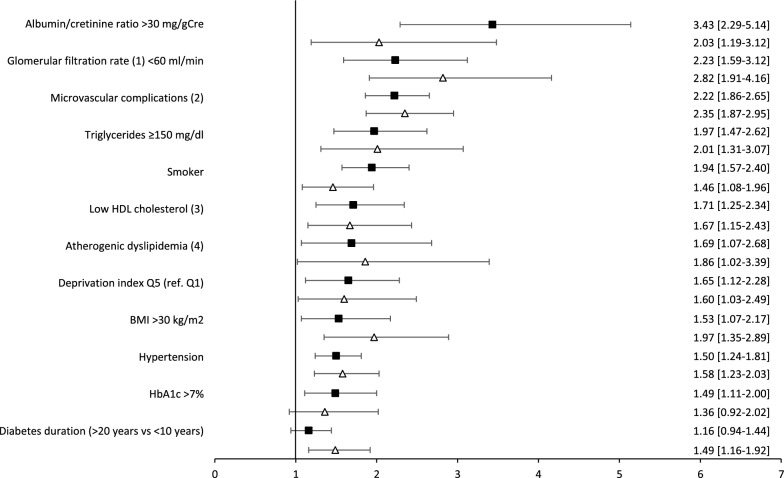
Age-adjusted hazard ratios for relevant baseline characteristics associated with a first cardiovascular event in men (bold squares) and women (open triangles) with type 1 diabetes. Glomerular filtration rate was according to CKD-EPI equation. Microvascular complications were according to ICD-10 diagnosis E10.2 to E10.4, N08.3, E36.0 or G63.2, glomerular filtration rate < 60 ml/min or albumin/creatinine ≥ 30 mg/g. Low HDL cholesterol: < 40 mg/dl in men and < 50 mg/dl in women. Atherogenic dyslipidaemia: triglycerides ≥ 150 mg/dl and low HDL cholesterol

The baseline characteristics of individuals with or without a CV event during the follow-up are shown in Additional file 1: Table S3. Most traditional CV risk factors were associated with an increased incidence of a CV event. Nonetheless, the strongest associations were observed for decreased glomerular filtration rate (HR 5.42 [95% CI 4.32;6.80]), elevated albumin/creatinine ratio (HR 3.39 [95% CI 2.47;4.66]), microvascular complications (HR 3.27 [95% CI 2.85;3.75]), and hypertension (HR 3.21 [95% CI 2.80;3.67]). In the age–adjusted analyses, the relationship between baseline characteristics and incident first CV event were generally similar in men and women, as shown by the similar the HRs and 95% CIs (Fig. 2). Treatment with anti-hypertensive drugs (HR 3.92 [95% CI 3.43;4.49], antiplatelet agents (HR 3.41 [95% CI 2.98;3.89], and statins (HR 2.30 [CI 2.02;2.63] also showed a strong association with CV events, remaining significant in the age-adjusted analysis in both men and women.

In the aged-stratified analysis, some differences emerged between age groups. Men were at higher risk of suffering a first CV event than women in all age groups except the Y and MVO groups. In addition, while in the EA group HRs were significant for all risk factors, some of them were not significant in the other groups. Again, the strongest associations were observed for renal disease and the presence of microvascular complications in all groups (Additional file 1: Table S4).

**Fig. 3:**
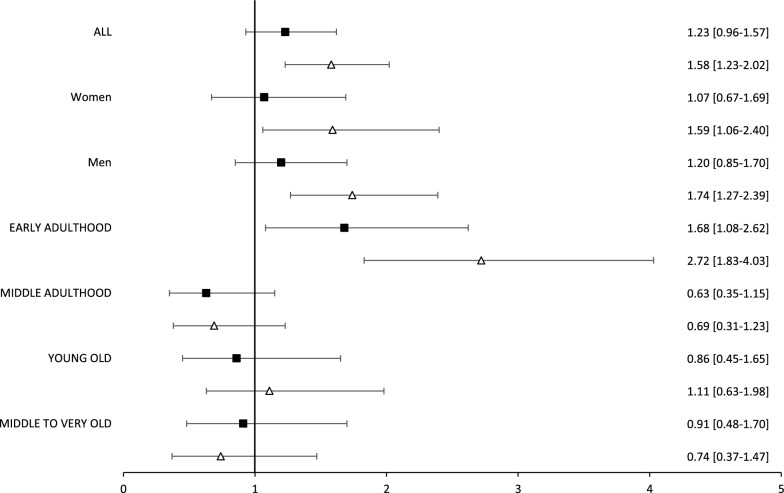
Aged-adjusted (whole population and according to sex) and age-stratified hazard ratios for HbA1c categories associated with a first cardiovascular event in patients with type 1 diabetes (bold squares HbA1c 7.0–8.0%; open triangles HbA1c > 8.0%; reference HbA1c < 7.0%) (Y group is not shown because there are no cases in the reference category)

When categorising HbA1c levels, we found that HbA1c 7–8%, and especially, HbA1c higher than 8%, were associated with a higher risk of a first CV event (Fig. 3, reference category HbA1c < 7%) in both men and women. This excess risk was mainly driven by the Y and EA groups, who represented roughly 84% of the study population. For the other age groups, HbA1c was not associated with an increased risk of a first CV event (Fig. 3)**.**

## Discussion

In this retrospective analysis of a large cohort of individuals with T1D, we have described the first manifestation of a CV event and provided incidence rates according to sex and age. Importantly, we observed that (1) peripheral artery disease is the most frequent first-ever CV event, and (2) that age and sex modulate not only the overall CV risk, but also the form of initial CV disease presentation.

We observed an incidence rate (per 100 PYs) of a first CVD event of 1.62, similar to that of a contemporary study in Scotland [21] with a rate of 1.4 in 27,527 individuals older than 18 years and a follow-up of 199,552 PYs. Conversely, our results yielded a much higher incidence than a Finnish study [22] with 1761 individuals with an event with a follow-up of 361,033 PYs during a median of 29.6 years. This variability may be due to differences in the overall design, the inclusion criteria, and the method of data retrieval. However, it is important to highlight that both studies, like ours, included peripheral artery disease as an outcome and, therefore are quite comparable. Comparison with other studies is more difficult since, generally, peripheral artery disease [6] and in some cases also heart failure [23, 24], have not been included as outcomes when analysing the incidence and factors associated with CVD in patients with T1D. We consider that including both peripheral artery disease and heart failure as CV outcomes provides a clearer and more comprehensive picture of the CV burden in T1D. Indeed, we observed a high rate of peripheral artery disease as a first CV event, and a preponderance of HF at older ages, especially in women, and these findings reinforce the importance of considering these outcomes in clinical practice and risk estimation. Overall, taking all these considerations into account, it can be assumed that the incidence of CVD in patients with T1D in Catalonia, a region considered to be of low CV risk [25], is not lower to that of other European areas, in contrast to what occurs in the general non-diabetic population. This finding suggests that type 1 diabetes may override the protective geographic effect.

When comparing the rates of the different types of first CV events with other studies, our results are similar to those reported by Harjustsalo [22] in terms of the number of coronary and peripheral vascular events; however, they are clearly different from other studies showing a preponderance of ischemic heart events [21, 26] over peripheral vascular events. In addition, we observed some variations in the type of the first manifestation of CVD by sex and age*,* data that, to the best of our knowledge, have not been reported in other studies. While atherosclerotic events, mainly peripheral artery disease and coronary heart disease, were the most common presentations of CVD in males of all ages and young women, there was a marked increase in the proportion of heart failure events in older women beginning at the age of 60.

Regarding the risk factors, it is worth noting the strong association between a first CV event and the presence of renal variables and microvascular complications and hypertension, as previously shown in other studies [23, 24]. Higher HbA1c levels have been consistently linked with CVD risk in people with T1D [9, 22, 23, 27], although in some cases this relationship was attenuated by the duration of diabetes [24]. In our study, this relationship was highly dependent on age, being clear in young patients, and losing significance in patients older than 55–60 years. In this regard, the association between higher HbA1c and subsequent CVD events has been shown to be increasingly mediated by its effect on standard risk factors as patients become older [11], reinforcing the importance of managing traditional non-glycaemic CVD risk factors in an ageing T1D population with longstanding hyperglycaemia. However, as in other studies [21, 24], we found that treatment of these risk factors with statins, and hypotensive and antiplatelet agents were positively associated with a first CV event, most probably reflecting that although high-risk patients are adequately identified, prevention strategies are not always sufficient to completely lower their risk.

All these data may have important clinical implications. First, they could help to guide the evaluation and discussion of CV risks and preventive strategies in the clinical setting and might also facilitate shared medical decisions from a more individualised perspective. Second, they can help outline the efficacy of current CV preventive strategies in people with T1D throughout their lives and define new strategies if necessary. It should be noted that the CV risk management in people with T1D has mainly been extrapolated from data obtained in people with type 2 diabetes [28], with proven efficacy in reducing the CV morbidity and mortality [4]. However, the CV burdens of T1D might be further reduced by considering sex and age-specific risks and modulating medical interventions accordingly. In this sense, it could be important to consider a stricter approach to the treatment of hypertension in older individuals with T1D, especially women, in view of the high incidence of HF in this population and the beneficial effects of intensive treatment of hypertension in the general population [29]. Unfortunately, prevention strategies for peripheral artery disease, an important component of CV morbidity in patients with T1D, are less established, but in any case, its high prevalence should prompt awareness of the need for early detection in asymptomatic patients and, perhaps, earlier initiation of combined antithrombotic therapy [28].

## Limitations

Our study has several limitations. First, we used diagnoses available through a primary-care-based electronic record database without external validation measures. Thus, we cannot exclude some inter-individual variability in disease definitions and the possibility of underreporting of diseases not directly supervised in primary care. Second, T1D duration was shorter when compared to most studies, and not clearly different between age groups. This is a matter of concern, as it might be due to misdiagnosis between T1D and type 2 diabetes. However, we applied restrictive criteria to assign a T1D diagnosis and therefore misdiagnosis is unlikely. We believe that it is more likely due to errors in the assigned date of diagnosis of the disease. The primary-care clinical station, which is a main component of the SIDIAP database, allowed as the date of diagnosis the first time a diagnosis is used in the clinical station. This problem was detected for cases that were initially included at the time of the implementation of the electronic clinical records in 2005. This fact may mean that, in some cases, especially those with a diagnosis before the implementation of the electronic clinical station (i.e., with longer diabetes duration), the date of diagnosis was not correctly recorded. Third, for some variables (including BMI, blood pressure, and laboratory parameters), there was a substantial proportion of missing values. Therefore, the analysis assessing the association between risk factors and CVD should be interpreted with caution. Finally, causes of death were not available, and therefore cases of sudden cardiac death occurring outside the hospital could not be analysed. However, more than 50% of sudden cardiac deaths in type 1 diabetes occur in patients with previously known coronary heart disease or atrial fibrillation [30] that are exclusion criteria of our study.

In conclusion, peripheral artery disease and, in the elderly, heart failure contributes significantly to the burden of CVD in people with T1D. This epidemiological evidence should be considered when introducing primary prevention measures and when estimating CV risk and could be relevant in the design of future clinical trials.

## Supplementary Information

**Additional file 1:** **Table S1.** ICD-10 codes of cardiovascular disease. **Table S2.** Clinical characteristics of patients according to age groups. Data are mean [SD] for quantitative variables and n (%) for categorical variables. ^1^ICD-10 diagnosis E10.2 to E10.4, N08.3, E36.0, G63.2, glomerular filtration rate < 60 ml/min or albumin/creatinine ≥ 30 mg/g. **Table S3.** Baseline characteristics according to the cardiovascular event status at follow-up. Data are mean [SD] for quantitative variables, and n (%) for categorical variables. ^1^HDL-cholesterol < 40 mg/dl in men and < 50 mg/dl in women. ^2^Triglycerides ≥ 150 mg/dl and low HDL cholesterol. ^3^ICD-10 diagnosis E10.2 to E10.4, N08.3, E36.0 or G63.2. Age groups: young (Y): < 35 years; early adulthood (EA): 35 to 55/60 (men/women) years; middle adulthood (MA): 55/60 to 65 (men/women); young old (YO): 66 to 75 years and middle-to-very old (MVO): > 75 years. **Table S4.** Baseline characteristics by age category according to the cardiovascular event status at follow-up. Data are mean [SD] for quantitative variables, and n (%) for categorical variables. ^1^HDL-cholesterol < 40 mg/dl in men and < 50 mg/dl in women. ^2^Triglycerides ≥ 150 mg/dl and low HDL cholesterol. ^3^ICD-10 diagnosis E10.2 to E10.4, N08.3, E36.0 or G63.2. Age groups: young (Y): < 35 years; early adulthood (EA): 35 to 55/60 (men/women) years; middle adulthood (MA): 55/60 to 65 (men/women); young old (YO): 66 to 75 years and middle-to-very old (MVO): > 75 years. **Table S5.** Age and sex distribution of participants who died versus those who did not die at the end of follow-up. Age groups: young (Y): < 35 years; early adulthood (EA): 35 to 55/60 (men/women) years; middle adulthood (MA): 55/60 to 65 (men/women); young old (YO): 66 to 75 years and middle-to-very old (MVO): > 75 years. **Table S6.** Age and sex distribution of deceased participants according to the absence/presence of a first cardiovascular event during the study period. Age groups: young (Y): < 35 years; early adulthood (EA): 35 to 55/60 (men/women) years; middle adulthood (MA): 55/60 to 65 (men/women); young old (YO): 66 to 75 years and middle-to-very old (MVO): > 75 years.

## Data Availability

The datasets used and/or analyzed during the current study are available from the corresponding author on reasonable request.

## Abbreviations

BMI: Body mass index
BMSD: Basic minimum set of data
CI: Confidence intervals
CKD-EPI: Chronic Kidney Disease Epidemiology Collaboration
CV: Cardiovascular
CVD: Cardiovascular disease
EA: Early adulthood (35 to 55/60 (men/women) years)
EGFR: Estimated glomerular filtration rate
HR: Hazard ratio
ICD-10: International Classification of Diseases 10
ICS: Institut Català de la Salut (Catalan Institute of Health)
IQR: Interquartile range
MA: Middle adulthood (55/60 to 65 (men/women) years)
MI: Myocardial infarction
MVO: Middle-to-very old (> 75 years)
PY: Persons-years
SD: Standard deviation
SIDIAP: Sistema d'Informació per al desenvolupament de la Investigació en Atenció Primària. (Information System for the Development of Primary Care Research)
T1D: Type 1 diabetes
Y: Young (< 35 years)
YO: Young old (66 to 75 years)
