# Supplementary material for: Epidemiology of the first-ever cardiovascular event in people with type 1 diabetes: a retrospective cohort population-based study in Catalonia
Source: Cardiovasc Diabetol. 2023 Jul 14;22:179. doi: 10.1186/s12933-023-01917-1 (PMC10349453; doi:10.1186/s12933-023-01917-1)
Supplement: Supplementary file 1 — Additional file 1: Table S1. ICD-10 codes of cardiovascular disease. Table S2. Clinical characteristics of patients according to age groups. Data are mean [SD] for quantitative variables and n (%) for categorical variables. 1ICD-10 diagnosis E10.2 to E10.4, N08.3, E36.0, G63.2, glomerular filtration rate < 60 ml/min or albumin/creatinine ≥ 30 mg/g. Table S3. Baseline characteristics according to the cardiovascular event status at follow-up. Data are mean [SD] for quantitative variables, and n (%) for categorical variables. 1HDL-cholesterol < 40 mg/dl in men and < 50 mg/dl in women. 2Triglycerides ≥ 150 mg/dl and low HDL cholesterol. 3ICD-10 diagnosis E10.2 to E10.4, N08.3, E36.0 or G63.2. Age groups: young (Y): < 35 years; early adulthood (EA): 35 to 55/60 (men/women) years; middle adulthood (MA): 55/60 to 65 (men/women); young old (YO): 66 to 75 years and middle-to-very old (MVO): > 75 years. Table S4. Baseline characteristics by age category according to the cardiovascular event status at follow-up. Data are mean [SD] for quantitative variables, and n (%) for categorical variables. 1HDL-cholesterol < 40 mg/dl in men and < 50 mg/dl in women. 2Triglycerides ≥ 150 mg/dl and low HDL cholesterol. 3ICD-10 diagnosis E10.2 to E10.4, N08.3, E36.0 or G63.2. Age groups: young (Y): < 35 years; early adulthood (EA): 35 to 55/60 (men/women) years; middle adulthood (MA): 55/60 to 65 (men/women); young old (YO): 66 to 75 years and middle-to-very old (MVO): > 75 years. Table S5. Age and sex distribution of participants who died versus those who did not die at the end of follow-up. Age groups: young (Y): < 35 years; early adulthood (EA): 35 to 55/60 (men/women) years; middle adulthood (MA): 55/60 to 65 (men/women); young old (YO): 66 to 75 years and middle-to-very old (MVO): > 75 years. Table S6. Age and sex distribution of deceased participants according to the absence/presence of a first cardiovascular event during the study period. Age groups: young (Y): < 35 years; early adulth [file 12933_2023_1917_MOESM1_ESM.docx]

Additional file for “Epidemiology of the first-ever cardiovascular event in people with type 1 diabetes: a retrospective cohort population-based study in Catalonia”

Short title: Cardiovascular events in type 1 diabetes

**Authors**: Gabriel Giménez-Pérez MD, PhD^1,2^, Clara Viñals MD, PhD^3^, Manel Mata-Cases MD, PhD^4,5,6^, Bogdan Vlacho PhD^4,5^, Jordi Real PhD^4,5^, Josep Franch-Nadal MD, PhD^4,5,7^, , Emilio Ortega MD, PhD^3,4,8^, Dídac Mauricio MD, PhD^4,5,9,10^

**Affiliations**:

^1^Section of Endocrinology, Department of Medicine, Hospital General de Granollers, Granollers, Spain.

^2^School of Medicine and Health Sciences, Universitat Internacional de Catalunya, Sant Cugat del Vallès, Spain.

^3^Department of Endocrinology & Nutrition, Hospital Clínic de Barcelona. Barcelona, Spain.

^4^DAP-Cat Group. Unitat de Suport a La Recerca Barcelona Ciutat, Institut Universitari d'Investigació en Atenció Primària Jordi Gol. Barcelona, Spain.

^5^CIBER of Diabetes and Associated Metabolic Diseases (CIBERDEM), Instituto de Salud Carlos III (ISCIII), Barcelona, Spain.

^6^Primary Health Care Center La Mina, Gerència d'Àmbit d'Atenció Primària Barcelona Ciutat, Institut Català de La Salut, Sant Adrià de Besòs, Spain.

^7^Primary Health Care Center Raval Sud, Gerència d'Àmbit d'Atenció Primària Barcelona Ciutat, Institut Català de La Salut, Barcelona, Spain.

^8^CIBER of Physiopathology of Obesity and Nutrition, ISCIII, Madrid, Spain.

^9^Department of Endocrinology & Nutrition, Hospital de la Santa Creu i Sant Pau, Barcelona, Spain.

^10^Department of Medicine, University of Vic - Central University of Catalonia, Vic, Spain.

**Supplementary Table 1.** ICD-10 codes of cardiovascular disease

**Supplementary Table 2.** Clinical characteristics of patients according to age groups

**Supplementary Table 3.** Baseline characteristics according to the cardiovascular event status at follow-up

**Supplementary Table 4.** Baseline characteristics by age category according to the cardiovascular event status at follow-up

**Supplementary Table 5.** Age and sex distribution of participants who died versus those who did not die at the end of follow-up.

**Supplementary Table 6.** Age and sex distribution of deceased participants according to the absence/presence of a first cardiovascular event during the study period

**Supplementary Table 1**

ICD-10 codes of cardiovascular disease

| ICD-10 code | Description | Type of cardiovascular event |
| --- | --- | --- |
| I20.* | Angina pectoris | Coronary |
| I20.0 | Unstable angina | Coronary |
| I20.1 | Angina pectoris with documented spasm | Coronary |
| I20.8 | Other forms of angina pectoriS | Coronary |
| I20.9 | Angina pectoris, unspecified | Coronary |
| I21* | Acute myocardial infarction | Coronary |
| I21.0 | Acute transmural myocardial infarction of anterior wall | Coronary |
| I21.1 | Acute transmural myocardial infarction of inferior wall | Coronary |
| I21.2 | Acute transmural myocardial infarction of other sites | Coronary |
| I21.3 | Acute transmural myocardial infarction of unspecified site | Coronary |
| I21.4 | Acute subendocardial myocardial infarction | Coronary |
| I21.9 | Acute myocardial infarction, unspecified | Coronary |
| I22* | Subsequent myocardial infarction | Coronary |
| I22.0 | Subsequent myocardial infarction of anterior wall | Coronary |
| I22.1 | Subsequent myocardial infarction of inferior wall | Coronary |
| I22.8 | Subsequent myocardial infarction of other sites | Coronary |
| I22.9 | Subsequent myocardial infarction of unspecified site | Coronary |
| I23* | Certain current complications following acute myocardial infarction | Coronary |
| I23.0 | Haemopericardium as current complication following acute myocardial infarction | Coronary |
| I23.1 | Atrial septal defect as current complication following acute myocardial infarction | Coronary |
| I23.2 | Ventricular septal defect as current complication following acute myocardial infarction | Coronary |
| I23.3 | Rupture of cardiac wall without haemopericardium as current complication following acute myocardial infarction | Coronary |
| I23.4 | Rupture of chordae tendineae as current complication following acute myocardial infarction | Coronary |
| I23.5 | Rupture of papillary muscle as current complication following acute myocardial infarction | Coronary |
| I23.6 | Thrombosis of atrium, auricular appendage, and ventricle as current complications following acute myocardial infarction | Coronary |
| I23.8 | Other current complications following acute myocardial infarction | Coronary |
| I24 | Other acute ischaemic heart diseases | Coronary |
| I24.0 | Coronary thrombosis not resulting in myocardial infarction | Coronary |
| I24.1 | Dressler syndrome | Coronary |
| I24.8 | Other forms of acute ischaemic heart disease | Coronary |
| I24.9 | Acute ischaemic heart disease, unspecified | Coronary |
| I25 | Chronic ischaemic heart disease | Coronary |
| I25.0 | Atherosclerotic cardiovascular disease, so described | Coronary |
| I25.1 | Atherosclerotic heart disease | Coronary |
| I25.2 | Old myocardial infarction | Coronary |
| I25.3 | Aneurysm of heart | Coronary |
| I25.4 | Coronary artery aneurysm and dissection | Coronary |
| I25.5 | Ischaemic cardiomyopathy | Coronary |
| I25.6 | Silent myocardial ischaemia | Coronary |
| I25.8 | Other forms of chronic ischaemic heart disease | Coronary |
| I25.9 | Chronic ischaemic heart disease, unspecified | Coronary |
| I61* | Intracerebral haemorrhage | Cerebrovascular |
| I63* | Cerebral infarction | Cerebrovascular |
| I65* | Occlusion and stenosis of precerebral arteries, not resulting in cerebral infarction | Cerebrovascular |
| G45* | Transient cerebral ischaemic attacks and related syndromes | Cerebrovascular |
| G46* | Vascular syndromes of brain in cerebrovascular diseases | Cerebrovascular |
| I65* | Occlusion and stenosis of precerebral arteries, not resulting in cerebral infarction | Peripheral vascular |
| I70.2 | Atherosclerosis of arteries of extremities | Peripheral vascular |
| I73.9 | Peripheral vascular disease, unspecified | Peripheral vascular |
| I11.0 | Hypertensive heart disease with (congestive) heart failure | Heart failure |
| I13.0 | Hypertensive heart and renal disease with (congestive) heart failure | Heart failure |
| I50 | Heart failure | Heart failure |

**Supplementary Table 2. Clinical characteristics of patients according to age groups**

|  | ALL | Y | EA | MA | YO | MVO | p.trend | N |
| --- | --- | --- | --- | --- | --- | --- | --- | --- |
|  | N=8412 | N=1810 (21.5) | N=5231 (62.2) | N=696 (8.3) | N=411 (4.9) | N=264 (3.1) |  |  |
| Age (years) | 42.1 (12.1) | 32.5 (1.46) | 44.0 (6.24) | 59.9 (2.82) | 69.2 (2.83) | 80.5 (3.98) | 0.000 | 8412 |
| Sex: Women | 3559 (42.3) | 737 (40.7) | 2283 (43.6) | 161 (23.1) | 211 (51.3) | 167 (63.3) | 0.001 | 8412 |
| Diabetes duration (years) | 10.9 (10) | 8.93 (7.13) | 11.0 (9.6) | 13.8 (13) | 13.7 (14.9) | 11.0 (13.2) | <0.001 | 8412 |
| Smoking: |  |  |  |  |  |  | 0.000 | 6858 |
| No smoker | 3271 (47.7) | 605 (42.3) | 1928 (44.8) | 318 (56.0) | 248 (72.1) | 172 (81.5) |  |  |
| Smoker | 2654 (38.7) | 663 (46.4) | 1774 (41.2) | 151 (26.6) | 50 (14.5) | 16 (7.58) |  |  |
| Former smoker | 933 (13.6) | 162 (11.3) | 603 (14.0) | 99 (17.4) | 46 (13.4) | 23 (10.9) |  |  |
| Systolic Blood Pressure (mmHg) | 128 (15.7) | 122 (13.5) | 127 (14.9) | 133 (15.7) | 136 (17.0) | 139 (18.3) | <0.001 | 4419 |
| Diastolic Blood Pressure (mmHg) | 74 (9) | 73 (9) | 75 (9) | 74 (9) | 72 (10) | 70 (10) | <0.001 | 4419 |
| Body mass index (BMI) Kg/m^2^ | 25.6 (4.05) | 24.8 (3.97) | 25.6 (4.03) | 25.7 (3.78) | 26.4 (4.29) | 27.0 (4.20) | <0.001 | 3121 |
| BMI (categorized) |  |  |  |  |  |  | 0.001 | 3121 |
| <30 kg/m^2^ | 2736 (87.7) | 513 (90.8) | 1635 (87.5) | 285 (88.8) | 188 (82.5) | 115 (82.7) |  |  |
| ≥30 kg/m^2^ | 385 (12.3) | 52 (9.20) | 233 (12.5) | 36 (11.2) | 40 (17.5) | 24 (17.3) |  |  |
| Total cholesterol (mg/dL) | 192(37.0) | 186 (36.3) | 194 (37.0) | 190 (38.7) | 190 (35) | 187 (36.6) | 0.986 | 3299 |
| HDL (mg/dL) | 59.6 (17.1) | 57.2 (15.9) | 60.3 (17.0) | 61.0 (18.0) | 60.6 (18.9) | 56.5 (17.3) | 0.391 | 2919 |
| LDL (mg/dL) | 113 (30.6) | 110 (30.7) | 115 (30.4) | 111 (32.1) | 108 (29.2) | 110 (29.8) | 0.219 | 2919 |
| Triglyceride (mg/dL) | 108 (84.4) | 104 (69.9) | 109 (94.0) | 104 (69.3) | 108 (64.5) | 110 (54.5) | 0.604 | 2786 |
| Non-HDL cholesterol (mg/dL) | 132 (34.5) | 128 (34.8) | 134 (34.3) | 128 (35.1) | 129 (33.2) | 131 (34.6) | 0.801 | 2919 |
| Remnant-C (TC-HDLc-LDLc) (mg/dL) | 18.8 (10.8) | 18.0 (10.2) | 18.8 (11.2) | 18.3 (9.6) | 20.4 (10.1) | 21.1 (10.2) | 0.017 | 2919 |
| HbA1c (%) | 7.87 (1.64) | 7.90 (1.88) | 7.91 (1.60) | 7.81 (1.47) | 7.82 (1.52) | 7.43 (1.41) | 0.006 | 3245 |
| HbA1c (categorized): |  |  |  |  |  |  | 0.059 | 3245 |
| <7.0% | 932 (28.7) | 199 (31.7) | 550 (28.0) | 71 (24.1) | 58 (26.9) | 54 (37.5) |  |  |
| 7.0-8.0% | 955 (29.4) | 162 (25.8) | 569 (28.9) | 111 (37.6) | 65 (30.1) | 48 (33.3) |  |  |
| >8.0% | 1358 (41.9) | 266 (42.5) | 844 (43.1) | 113 (38.3) | 93 (43.0.1) | 42 (29.2) |  |  |
| Albumin/Creatinine (mg/g) | 35.0 (168) | 28.4 (136) | 37.6 (187) | 36.2 (140) | 28.2 (115) | 33.9 (115) | 0.252 | 1731 |
| Albumin/Creatinine (categorized) |  |  |  |  |  |  | <0.001 | 1731 |
| <30 mg/g | 1550 (89.5) | 284 (92.8) | 967 (90.0) | 139 (87.4) | 105 (86.8) | 55 (78.6) |  |  |
| ≥30 mg/g | 181 (10.5) | 22 (7.2) | 108 (10.0) | 20 (12.6) | 16 (13.2) | 15 (21.4) |  |  |
| Glomerular Filtration rate (ml/min/1.73m^2^) | 83.3 (13.6) | 88.1 (7.37) | 85.1 (11.3) | 80.7 (14.7) | 72.5 (18.4) | 61.5 (13.6) | <0.001 | 3268 |
| Glomerular Filtration rate (categorized) |  |  |  |  |  |  | 0.000 | 3268 |
| <60 ml/min/1.73m^2^ | 235 (7.19) | 10 (1.62) | 82 (4.15) | 27 (8.94) | 46 (21.0) | 70 (45.2) |  |  |
| ≥60 ml/min/1.73m^2^ | 3033 (92.8) | 606 (98.4) | 1894 (95.9) | 275 (91.1) | 173 (79.0) | 85 (54.8) |  |  |
| Microvascular complications^1^ (yes) | 1411 (16.8) | 165 (9.12) | 838 (16.0) | 205 (29.5) | 124 (30.2) | 79 (29.9) | 0.000 | 8412 |
| Statin treatment (yes) | 2479 (29.5) | 197 (10.9) | 1562 (29.9) | 362 (52.0) | 223 (54.3) | 135 (51.1) | 0.000 | 8412 |
| Anti-hypertensive drug treatment (yes) | 2540 (30.2) | 220 (12.2) | 1409 (26.9) | 391 (56.2) | 302 (73.5) | 218 (82.6) | 0.000 | 8412 |
| Antiplatelet treatment (yes) | 1834 (21.8) | 61 (3.37) | 1094 (20.9) | 316 (45.4) | 218 (53.0) | 145 (54.9) | 0.000 | 8412 |
| Short-acting insulin (yes) | 7256 (86.3) | 1690 (93.4) | 4661 (89.1) | 530 (76.1) | 263 (64.0) | 112 (42.4) | 0.000 | 8412 |
| Long-acting insulin analogs (yes) | 5896 (70.1) | 1375 (76.0) | 3740 (71.5) | 444 (63.8) | 222 (54.0) | 115 (43.6) | 0.000 | 8412 |
| NPH insulin mixture (yes) | 1910 (22.7) | 347 (19.2) | 1177 (22.5) | 184 (26.4) | 140 (34.1) | 62 (23.5) | <0.001 | 8412 |
| NPH insulin (yes) | 1533 (18.2) | 282 (15.6) | 881 (16.8) | 153 (22.0) | 106 (25.8) | 111 (42.0) | 0.000 | 8412 |
| Deprivation index (medea quintiles): |  |  |  |  |  |  | <0.001 | 6098 |
| Q1 | 1342 (22.0) | 233 (17.7) | 850 (22.1) | 138 (28.3) | 73 (25.1) | 48 (31.2) |  |  |
| Q2 | 1286 (21.1) | 273 (20.8) | 810 (21.0) | 108 (22.1) | 58 (19.9) | 37 (24.0) |  |  |
| Q3 | 1314 (21.5) | 304 (23.2) | 800 (20.8) | 101 (20.7) | 76 (26.1) | 33 (21.4) |  |  |
| Q4 | 1193 (19.9) | 271 (20.6) | 767 (19.9) | 86 (17.6) | 49 (16.8) | 20 (13.0) |  |  |
| Q5 | 963 (15.8) | 234 (17./) | 623 (16.2) | 55 (11.3) | 35 (12.0) | 16 (10.4) |  |  |

Data are mean [SD] for quantitative variables, and n (%] for categorical variables. ^1^ICD-10 diagnosis E10.2 to E10.4, N08.3, E36.0, G63.2, glomerular filtration rate < 60ml/min or albumin/creatinine ≥30mg/g.

**Supplementary Table 3**

**Baseline characteristics according to the cardiovascular event status at follow-up**

|  | No event | Event | HR |
| --- | --- | --- | --- |
|  | n=7528 | n=884 |  |
| Age (Years) | 44.0 (11.3) | 55.4 (13.8) | 1.06 [1.06;1.07] |
| Sex |  |  |  |
| Women | 3232 (42.9%) | 327 (37.0%) | Ref. |
| Men | 4296 (57.1%) | 557 (63.0%) | 1.27 [1.11;1.45] |
| Age group |  |  |  |
| YO | 1774 | 36 | 0.21 [0.15;0.29] |
| EA | 4572 | 479 | Ref. |
| MA | 548 | 148 | 2.24 [1.90;2.65] |
| YO | 288 | 123 | 3.16 [2.66;3.74] |
| MVO | 166 | 9 | 3.74 [3.10;4.50] |
| Diabetes duration (years) | 10.7 (9.61) | 13.1 (12.9) | 1.02 [1.01;1.03] |
| Diabetes duration (categorical) |  |  |  |
| <10 years | 4963 (65.9%) | 545 (61.7%) | Ref. |
| 10-20 years | 1398 (18.6%) | 138 (15,6%) | 0.90 [0.76;1.09] |
| >20 years | 1167 (15.5%) | 201 (22.7%) | 1.51 [1.29;1.78] |
| Smoking: |  |  |  |
| No smoker | 2942 (48.1%) | 329 (44.2%) | Ref. |
| Smoker (current or previous) | 3172 (51.9%) | 415 (55.8%) | 1.15 [1.01;1.32] |
| HbA1c (%) | 7.82 (1.60) | 8.26 (1.83) | 1.14 [1.09;1.20] |
| HbA1c (categorical) |  |  |  |
| <7% | 841 (29.7%) | 91 (22.2%) | Ref. |
| ≥7% | 1995 (70.3%) | 318 (77.8%) | 1.43 [1.14;1.81] |
| Hypertension |  |  |  |
| No | 6282 (83.4%) | 530 (60.0%) | Ref. |
| Yes | 1246 (16.6%) | 354 (40.0%) | 3.21 [2.80;3.67] |
| Systolic blood pressure (mmHg) | 127 (15.0) | 135 (18.1) | 1.03 [1.02;1.03] |
| Diastolic Blood Pressure (mmHg) | 74.3 (8.95) | 74.6 (10.6) | 1.00 [0.99;1.01] |
| Body mass index (kg/m^2^) | 25.5 (3.93) | 26.3 (4.78) | 1.04 [1.02;1.07] |
| Body mass index (categorical) |  |  |  |
| <30 kg/m2 | 2443 (88.7%) | 293 (79.8%) | Ref. |
| ≥30 kg/m2 | 311 (11.3%) | 74 (20.2%) | 1.85 [1.43;2.39] |
| Total cholesterol (mg/dl) per 10 mg/dl units | 19.2 (3.66) | 19.2 (4.02) | 1.00 [0.97;1.02] |
| HDL-cholestrol (mg/dl) per 10 mg/dl units | 6.04 (1.71) | 5.43 (1.59) | 0.80 [0.75;0.86] |
| LDL-cholesterol (mg/dl) per 10 mg/dl units | 11.3 (3.00) | 11.3 (3.45) | 1.00 [0.96;1.03] |
| HDL cholesterol (categorical) |  |  |  |
| Normal/High | 2188 (85.9%) | 284 (76.5%) | Ref. |
| Low^1^ | 360 (14.1%) | 87 (23.5%) | 1.78 [1.40;2.27] |
| Triglyceride (mg/dl) per 10 mg/dl units | 10.4 (8.03) | 13.2 (10.4) | 1.019 [1.01;1.02] |
| Triglyceride (categorical) |  |  |  |
| <150 mg/dl | 2105 (87.3%) | 284 (75.5%) | Ref. |
| ≥ 150 mg/dl | 305 (12.7%) | 92 (24.5%) | 2.10 [1.66;2.65] |
| Non-HDL cholesterol per 10 mg/dl units | 13.1 (3.36) | 13.6 (3.95) | 1.04 [1.01;1.07] |
| Remnant-C (TC-HDLc-LDLc) per 5 mg/dl units | 3.64 (2.06) | 4.66 (2.54) | 1.16 [1.12;1.20] |
| Atherogenic dyslipidemia^2^ |  |  |  |
| No | 2194 (95.7%) | 325 (91.0%) | Ref. |
| Yes | 99 (4.32%) | 32 (8.96%) | 2.06 [1.43;2.96] |
| Glomerular Filtration (CKD-EPI; ml/min 1,73 m2) | 84.8 (11.4) | 73.6 (21.0) | 0.96 [0.96;0.97] |
| CKD-EPI (categorical) |  |  |  |
| ≥60 ml/min 1,73 m^2^ | 2709 (95.2%) | 324 (76.8%) | Ref. |
| <60 ml/min 1,73 m2 | 137 (4.81%) | 98 (23.2%) | 5.42 [4.32;6.80] |
| Albumin/Creatinine ratio (mg/gCre) | 26.8 (147) | 91.2 (321) | 1.00 [1.00;1.00] |
| Albumin/Creatinine ratio (categorical) |  |  |  |
| <30 mg/gCre | 1399 (91.5%) | 151 (74.8%) | Ref. |
| ≥30mg/gCre | 130 (8.50%) | 51 (25.2%) | 3.39 [2.47;4.66] |
| Microvascular complications^3^ |  |  |  |
| No | 6445 (85.6%) | 556 (62.9%) | Ref. |
| Yes | 1083 (14.4%) | 328 (37.1%) | 3.27 [2.85;3.75] |
| Deprivation index (MEDEA quintiles) |  |  |  |
| Q1 | 1221 (22.3%) | 121 (19.4%) | Ref. |
| Q2 | 1157 (21.1%) | 129 (20.6%) | 1.12 [0.87;1.43] |
| Q3 | 1177 (21.5%) | 137 (21.9%) | 1.16 [0.91;1.49] |
| Q4 | 1066 (19.5%) | 127 (20.3%) | 1.19 [0.93;1.53] |
| Q5 | 852 (15.6%) | 111 (17.8%) | 1.30 [1.01;1.68] |
| Statin treatment |  |  |  |
| No | 5469 (72.6%) | 464 (52.5%) | Ref. |
| Yes | 2059 (27.4%) | 420 (47.5%) | 2.30 [2.02;2.63] |
| Anti-hypertensive drug treatment |  |  |  |
| No | 5519 (73.3%) | 353 (39.9%) | Ref. |
| Yes | 2009 (26.7%) | 531 (60.1%) | 3.92 [3.43;4.49] |
| Antiplatelet drug treatment |  |  |  |
| No | 6101 (81.0%) | 477 (54.0%) | Ref. |
| Yes | 1427 (19.0%) | 407 (46.0%) | 3.41 [2.98;3.89] |

Data are mean [SD] for quantitative variables, and n (%] for categorical variables. ^1^HDL-cholesterol <40mg/dl in men and <50mg/dl in women. ^2^Triglycerides ≥150 mg/dl and low HDL cholesterol. ^3^ICD-10 diagnosis E10.2 to E10.4, N08.3, E36.0 or G63.2. Age groups: young (Y): <35 years; early adulthood (EA): 35 to 55/60 (men/women) years; middle adulthood (MA): 55/60 to 65 (men/women); young old (YO): 66 to 75 years and middle-to-very old (MVO): >75 years

**Supplementary Table 4**

**Baseline characteristics by age category according to the cardiovascular event status at follow-up** (Hazard ratios >1 in bold)

|  |  | Y |  |  | EA |  |  | MA |  |  | YO |  |  | MVO |  |
| --- | --- | --- | --- | --- | --- | --- | --- | --- | --- | --- | --- | --- | --- | --- | --- |
|  | No event | Event | HR | No event | Event | HR | No event | Event | HR | No event | Event | HR | No event | Event |  |
|  | N=1774 | N=36 |  | N=4752 | N=479 |  | N=548 | N=148 |  | N=288 | N=123 |  | N=166 | N=98 |  |
| Sex |  |  |  |  |  |  |  |  |  |  |  |  |  |  |  |
| Female | 722 (40.7%) | 15 (41.7%) | Ref. | 2107 (44.3%) | 176 (36.7%) | Ref. | 136 (24.8%) | 25 (16.9%) | Ref. | 158 (54.9%) | 53 (43.1%) | Ref. | 109 (65.7%) | 58 (59.2%) | Ref. |
| Male | 1052 (59.3%) | 21 (58.3%) | 0.96 [0.50;1.87] | 2645 (55.7%) | 303 (63.3%) | **1.35 [1.12;1.63]** | 412 (75.2%) | 123 (83.1%) | **1.63 [1.06;2.51**] | 130 (45.1%) | 70 (56.9%) | **1.53 [1.07;2.18]** | 57 (34.3%) | 40 (40.8%) | 1.35 [0.90;2.01] |
| Diabetes duration (years) | 8.87 (7.09) | 11.8 (8.63) | **1.05 [1.01;1.09]** | 10.9 (9.43) | 12.4 (11.2) | **1.01 [1.01;1.02]** | 13.8 (12.9) | 14.0 (13.7) | 1.00 [0.99;1.01] | 12.7 (13.6) | 16.0 (17.3) | 1.01 [1.00;1.02] | 10.5 (12.6) | 11.8 (14.2) | 1.00 [0.99;1.02] |
| DM1 duration (categoriacal) |  |  |  |  |  |  |  |  |  |  |  |  |  |  |  |
| <10 years | 1240 (69.9%) | 22 (61.1%) | Ref. | 3094 (65.1%) | 293 (61.2%) | Ref. | 317 (57.8%) | 90 (60.8%) | Ref. | 189 (65.6%) | 70 (56.9%) | Ref. | 123 (74.1%) | 70 (71.4%) | Ref. |
| 10-15 years | 218 (12.3%) | 4 (11.1%) | 1.03 [0.35;2.99] | 560 (11.8%) | 55 (11.5%) | 1.04 [0.78;1.38] | 53 (9.67%) | 12 (8.11%) | 0.81 [0.45;1.49] | 25 (8.68%) | 11 (8.94%) | 1.11 [0.59;2.09] | 15 (9.04%) | 4 (4.08%) | 0.44 [0.16;1.21] |
| 15-20 years | 145 (8.17%) | 4 (11.1%) | 1.55 [0.54;4.51] | 323 (6.80%) | 25 (5.22%) | 0.82 [0.55;1.24] | 39 (7.12%) | 8 (5.41%) | 0.72 [0.35;1.49] | 16 (5.56%) | 9 (7.32%) | 1.32 [0.66;2.65] | 4 (2.41%) | 6 (6.12%) | 1.51 [0.66;3.49] |
| >20 years | 171 (9.64%) | 6 (16.7%) | 1.95 [0.79;4.81] | 775 (16.3%) | 106 (22.1%) | **1.41 [1.13;1.76]** | 139 (25.4%) | 38 (25.7%) | 0.94 [0.64;1.37] | 58 (20.1%) | 33 (26.8%) | 1.39 [0.92;2.10] | 24 (14.5%) | 18 (18.4%) | 1.09 [0.65;1.83] |
| Smoking |  |  |  |  |  |  |  |  |  |  |  |  |  |  |  |
| No smoker | 597 (42.5%) | 8 (33.3%) | Ref. | 1786 (45.9%) | 142 (34.0%) | Ref. | 267 (59.2%) | 51 (43.6%) | Ref. | 178 (75.4%) | 70 (64.8%) | Ref. | 114 (85.1%) | 58 (75.3%) | Ref. |
| Smoker (current or former) | 709 (46.2%) | 16 (58.3%) | 1.67 [0.72;3.87] | 2101 (40.2%) | 276 (51.0%) | **1.58 [1.30;1.91]** | 184 (24.4%) | 66 (35.0%) | **1.65 [1.18;2.28]** | 58 (12.7%) | 38 (18.5%) | **1.40 [1.02;1.92]** | 20 (5.97%) | 19 (10.4%) | 1.45 [0.93;2.12] |
| HbA1c (%) | 7.85 (1.86) | 9.91 (1.68) | **1.38 [1.19;1.60]** | 7.83 (1.54) | 8.66 (1.88) | **1.30 [1.21;1.39]** | 7.84 (1.46) | 7.72 (1.51) | 0.95 [0.80;1.13] | 7.78 (1.49) | 7.92 (1.58) | 1.06 [0.91;1.23] | 7.50 (1.30) | 7.32 (1.59) | 0.91 [0.74;1.14] |
| HbA1c (categorical) |  |  |  |  |  |  |  |  |  |  |  |  |  |  |  |
| <7% | 199 (32.5%) | 0 (0.00%) | Ref. | 519 (29.5%) | 31 (15.0%) | Ref. | 50 (21.9%) | 21 (31.3%) | Ref. | 40 (27.2%) | 18 (26.1%) | Ref. | 33 (35.9%) | 21 (40.4%) | Ref. |
| ≥7% | 413 (67.5%) | 15 (100%) |  | 1238 (70.5%) | 175 (85.0%) | **2.30 [1.57;3.37]** | 178 (78.1%) | 46 (68.7%) | 0.66 [0.39;1.10] | 107 (72.8%) | 51 (73.9%) | 1.01 [0.59;1.72] | 59 (64.1%) | 31 (59.6%) | 0.83 [0.48;1.44] |
| Hypertension: |  |  |  |  |  |  |  |  |  |  |  |  |  |  |  |
| No | 1686 (95.0%) | 31 (86.1%) | Ref. | 4070 (85.6%) | 333 (69.5%) | Ref. | 333 (60.8%) | 81 (54.7%) | Ref. | 132 (45.8%) | 51 (41.5%) | Ref. | 61 (36.7%) | 34 (34.7%) | Ref. |
| Yes | 88 (4.96%) | 5 (13.9%) | **3.08 [1.20;7.91]** | 682 (14.4%) | 146 (30.5%) | **2.50 [2.06;3.04]** | 215 (39.2%) | 67 (45.3%) | 1.25 [0.90;1.72] | 156 (54.2%) | 72 (58.5%) | 1.17 [0.82;1.68] | 105 (63.3%) | 64 (65.3%) | 1.03 [0.68;1.56] |
| Systolic Blood Pressure (mmHg) | 122 (13.1) | 135 (21.9) | **1.06 [1.03;1.09]** | 126 (14.5) | 130 (17.0) | **1.02 [1.01;1.02]** | 132 (15.4) | 138 (16.3) | **1.02 [1.01;1.03]** | 135 (16.5) | 138 (17.9) | 1.01 [1.00;1.02] | 137 (17.1) | 142 (20.0) | 1.01 [1.00;1.02] |
| Diastolic Blood Pressure (mmHg) | 73.2 (8.85) | 79.9 (12.2) | **1.08 [1.03;1.13]** | 75.0 (8.80) | 76.6 (10.4) | **1.02 [1.01;1.03]** | 74.2 (9.02) | 73.8 (9.77) | 1.00 [0.97;1.02] | 72.2 (9.20) | 71.5 (10.1) | 0.99 [0.97;1.01] | 69.8 (9.44) | 70.6 (10.1) | 1.01 [0.99;1.04] |
| Body mass index (kg/m^2^) | 24.8 (3.94) | 24.6 (5.43) | 0.99 [0.86;1.14] | 25.5 (3.90) | 26.4 (5.13) | **1.05 [1.01;1.09]** | 25.8 (3.72) | 24.9 (3.95) | 0.94 [0.87;1.01] | 26.3 (4.18) | 26.6 (4.54) | 1.01 [0.95;1.06] | 26.5 (4.12) | 27.8 (4.26) | 1.05 [0.99;1.11] |
| Body mass index (categorical) |  |  |  |  |  |  |  |  |  |  |  |  |  |  |  |
| <30 kg/m^2^ | 502 (90.9%) | 11 (84.6%) | Ref. | 1502 (88.4%) | 133 (79.2%) | Ref. | 231 (88.8%) | 54 (88.5%) | Ref. | 129 (84.9%) | 59 (77.6%) | Ref. | 79 (87.8%) | 36 (73.5%) | Ref. |
| ≥30 kg/m^2^ | 50 (9.06%) | 2 (15.4%) | 1.81 [0.40;8.17] | 198 (11.6%) | 35 (20.8%) | **1.89 [1.30;2.74]** | 29 (11.2%) | 7 (11.5%) | 1.00 [0.46;2.20] | 23 (15.1%) | 17 (22.4%) | 1.38 [0.81;2.37] | 11 (12.2%) | 13 (26.5%) | 1.86 [0.98;3.50] |
| Total cholesterol (mg/dl) per 10 mg/dl units | 18.5 (3.58) | 21.7 (4.54) | **1.20 [1.07;1.35]** | 19.4 (3.64) | 19.6 (4.19) | 1.01 [0.97;1.05] | 19.2 (3.97) | 18.3 (3.47) | 0.94 [0.88;1.00] | 19.1 (3.41) | 18.8 (3.72) | 0.96 [0.90;1.03] | 18.6 (3.51) | 18.8 (3.95) | 1.01 [0.94;1.08] |
| LDL cholesterol (mg/dl) per 10 mg/dl units | 10.9 (3.03) | 13.7 (3.45) | **1.30 [1.11;1.52]** | 11.5 (2.97) | 11.5 (3.59) | 1.00 [0.95;1.05] | 11.2 (3.21) | 10.7 (3.19) | 0.94 [0.87;1.03] | 10.9 (2.80) | 10.8 (3.18) | 0.99 [0.91;1.08] | 10.9 (2.81) | 11.3 (3.30) | 1.04 [0.95;1.14] |
| HDL cholesterol (mg/dl) per 10 mg/dl units | 5.73 (1.59) | 5.42 (1.50) | 0.88 [0.60;1.27] | 6.09 (1.70) | 5.47 (1.59) | **0.79 [0.72;0.87]** | 6.27 (1.86) | 5.49 (1.39) | **0.79 [0.68;0.92]** | 6.33 (1.87) | 5.47 (1.82) | **0.77 [0.67;0.90]** | 5.91 (1.76) | 5.17 (1.56) | **0.78 [0.64;0.94]** |
| HDL colesterol (categorical) |  |  |  |  |  |  |  |  |  |  |  |  |  |  |  |
| Normal/high | 434 (84.6%) | 12 (92.3%) | Ref. | 1377 (86.4%) | 144 (77.0%) | Ref. | 182 (86.3%) | 49 (81.7%) | Ref. | 124 (88.6%) | 45 (70.3%) | Ref. | 71 (78.9%) | 34 (72.3%) | Ref. |
| Low^1^ | 79 (15.4%) | 1 (7.69%) | 0.46 [0.06;3.54] | 217 (13.6%) | 43 (23.0%) | **1.81 [1.29;2.55]** | 29 (13.7%) | 11 (18.3%) | 1.36 [0.71;2.62] | 16 (11.4%) | 19 (29.7%) | **2.50 [1.46;4.29]** | 19 (21.1%) | 13 (27.7%) | 1.34 [0.70;2.53] |
| Triglycerides (mg/dl) per 10 mg/dl units | 10.3 (6.99) | 13.5 (6.69) | 1.04 [0.99;1.09] | 10.4 (8.73) | 14.5 (13.1) | **1.02 [1.01;1.03]** | 10.3 (7.36) | 10.8 (5.23) | 1.01 [0.99;1.04] | 10.2 (6.11) | 12.3 (6.96) | **1.03 [1.01;1.06**] | 10.4 (4.53) | 12.2 (6.78) | **1.05 [1.01;1.10]** |
| Triglycerides (categorical) |  |  |  |  |  |  |  |  |  |  |  |  |  |  |  |
| <150 mg/dl | 413 (87.1%) | 8 (66.7%) | Ref. | 1305 (87.2%) | 138 (72.6%) | Ref. | 180 (87.8%) | 50 (83.3%) | Ref. | 128 (90.8%) | 53 (81.5%) | Ref. | 79 (84.0%) | 35 (71.4%) | Ref. |
| ≥ 150 mg/dl | 61 (12.9%) | 4 (33.3%) | **3.35 [1.01;11.1]** | 191 (12.8%) | 52 (27.4%) | **2.43 [1.77;3.34**] | 25 (12.2%) | 10 (16.7%) | 1.27 [0.65;2.51] | 13 (9.22%) | 12 (18.5%) | 1.83 [0.98;3.42] | 15 (16.0%) | 14 (28.6%) | **1.93 [1.04;3.60]** |
| Non-HDL cholesterol per 10 mg/dl units | 12.8 (3.41) | 16.3 (4.32) | **1.26 [1.11;1.43**] | 13.3 (3.35) | 13.9 (4.07) | **1.05 [1.01;1.09]** | 12.9 (3.5.2) | 12.6 (3.51) | 0.97 [0.90;1.04] | 12.7 (3.14) | 13.3 (3.69) | 1.04 [0.96;1.12] | 12.9 (3.18) | 13.6 (3.93) | 1.05 [0.97;1.13] |
| Remnant-C (TC-HDLc-LDLc) per 5 mg/dl units | 3.6 (2.0) | 5.1 (2.7) | **1.23 [1.06;1.43]** | 3.6 (2.1) | 4.8 (2.8) | **1.17 [1.12;1.23]** | 3.6 (1.9) | 3.9 (1.8) | 1.07  [0.95;1.21] | 3.8 (1.8) | 4.8 (2.4) | **1.17 [1.06;1.29]** | 4.1 (1.8) | 4.6 (2.4) | 1.12 [0.98;1.29] |
| Atherogenic dyslipidemia^2^: |  |  |  |  |  |  |  |  |  |  |  |  |  |  |  |
| No | 435 (96.5%) | 12 (100%) | Ref. | 1369 (95.7%) | 162 (90.0%) | Ref. | 182 (94.8%) | 51 (92.7%) | Ref. | 128 (96.2%) | 59 (93.7%) | Ref. | 80 (92.0%) | 41 (87.2%) | Ref. |
| Yes | 16 (3.55%) | 0 (0.00%) |  | 61 (4.27%) | 18 (10.0%) | **2.32 [1.43;3.78]** | 10 (5.21%) | 4 (7.27%) | 1.33 [0.48;3.69] | 5 (3.76%) | 4 (6.35%) | 1.42 [0.52;3.91] | 7 (8.05%) | 6 (12.8%) | 1.62 [0.69;3.82] |
| Glomerular Filtration (CKD-EPI; ml/min 1,73 m2) | 88.4 (6.45) | 78.7 (23.4) | 0.95 [0.93;0.98] | 85.9 (9.61) | 78.8 (19.3) | 0.97 [0.96;0.97] | 82.2 (12.6) | 75.7 (19.5) | 0.98 [0.96;0.99] | 74.5 (17.0) | 68.2 (20.6) | 0.98 [0.97;1.00] | 64.0 (19.3) | 57.2 (19.8) | 0.98 [0.97;0.99] |
| CKD-EPI (categorical) |  |  |  |  |  |  |  |  |  |  |  |  |  |  |  |
| ≥60 ml/min 1,73 m^2^ | 594 (98.7%) | 12 (85.7%) | Ref. | 1714 (97.1%) | 180 (85.7%) | Ref. | 218 (94.0%) | 57 (81.4%) | Ref. | 123 (83.1%) | 50 (70.4%) | Ref. | 60 (61.2%) | 25 (43.9%) | Ref. |
| <60 ml/min 1,73 m2 | 8 (1.33%) | 2 (14.3%) | **12.0 [2.67;53.4]** | 52 (2.94%) | 30 (14.3%) | **4.77 [3.24;7.02]** | 14 (6.03%) | 13 (18.6%) | **2.89 [1.58;5.28]** | 25 (16.9%) | 21 (29.6%) | **1.73 [1.04;2.88]** | 38 (38.8%) | 32 (56.1%) | **2.30 [1.36;3.89]** |
| Albumin/Creatinine ratio (mg/gCre) | 15.3 (65.1) | 29.7 (60.5) | 1.00 [1.00;1.01] | 29.1 (168) | 132 (431) | 1.00 [1.00;1.00] | 31.6 (111) | 55.5 (141) | 1.00 [1.00;1.00] | 20.3 (62.8) | 46.0 (128) | 1.00 [1.00;1.01] | 52.0 (238) | 47.6 (78.4) | 1.00 [1.00;1.00] |
| Albumin/Creatinine ratio (categorical) |  |  |  |  |  |  |  |  |  |  |  |  |  |  |  |
| <30 mg/gCre | 279 (93.0%) | 5 (83.3%) | Ref. | 891 (91.7%) | 76 (73.8%) | Ref. | 109 (90.1%) | 30 (78.9%) | Ref. | 79 (89.8%) | 26 (78.8%) | Ref. | 41 (85.4%) | 14 (63.6%) | Ref. |
| ≥30mg/gCre | 21 (7.00%) | 1 (16.7%) | 2.72 [0.32;23.3] | 81 (8.33%) | 27 (26.2%) | **3.56 [2.29;5.52]** | 12 (9.92%) | 8 (21.1%) | **2.53 [1.16;5.53]** | 9 (10.2%) | 7 (21.2%) | 2.01 [0.87;4.64] | 7 (14.6%) | 8 (36.4%) | **3.11 [1.28;7.55]** |
| Microvascular complications^3^: |  |  |  |  |  |  |  |  |  |  |  |  |  |  |  |
| No | 1624 (91.5%) | 21 (58.3%) | Ref. | 4078 (85.8%) | 315 (65.8%) | Ref. | 406 (74.1%) | 85 (57.4%) | Ref. | 220 (76.4%) | 67 (54.5%) | Ref. | 117 (70.5%) | 68 (69.4%) | Ref. |
| Yes | 150 (8.46%) | 15 (41.7%) | **7.43 [3.83;14.4]** | 674 (14.2%) | 164 (34.2%) | **3.00 [2.49;3.63]** | 142 (25.9%) | 63 (42.6%) | **1.87 [1.35;2.59]** | 68 (23.6%) | 56 (45.5%) | **2.30 [1.61;3.28]** | 49 (29.5%) | 30 (30.6%) | 1.03 [0.67;1.58] |
| Deprivation index (MEDEA quintiles) |  |  |  |  |  |  |  |  |  |  |  |  |  |  |  |
| Q1 | 230 (17.9%) | 3 (11.1%) | Ref. | 798 (22.8%) | 52 (14.9%) | Ref. | 108 (29.0%) | 30 (26.1%) | Ref. | 57 (27.3%) | 16 (19.5%) | Ref. | 28 (27.7%) | 20 (37.7%) | Ref. |
| Q2 | 269 (20.9%) | 4 (14.8%) | 1.14 [0.25;5.09] | 750 (21.4%) | 60 (17.2%) | 1.22 [0.84;1.78] | 75 (20.1%) | 33 (28.7%) | 1.44 [0.88;2.36] | 39 (18.7%) | 19 (23.2%) | 1.52 [0.78;2.95] | 24 (23.8%) | 13 (24.5%) | 0.92 [0.46;1.84] |
| Q3 | 298 (23.1%) | 6 (22.2%) | 1.56 [0.39;6.22] | 721 (20.6%) | 79 (22.7%) | **1.65 [1.16;2.34]** | 75 (20.1%) | 26 (22.6%) | 1.19 [0.70;2.01] | 60 (28.7%) | 16 (19.5%) | 0.88 [0.44;1.77] | 23 (22.8%) | 10 (18.9%) | 0.87 [0.41;1.86] |
| Q4 | 263 (20.4%) | 8 (29.6%) | 2.32 [0.61;8.73] | 689 (19.7%) | 78 (22.4%) | **1.71 [1.21;2.44]** | 70 (18.8%) | 16 (13.9%) | 0.85 [0.46;1.56] | 32 (15.3%) | 17 (20.7%) | 1.58 [0.80;3.12] | 12 (11.9%) | 8 (15.1%) | 1.31 [0.57;2.97] |
| Q5 | 228 (17.7%) | 6 (22.2%) | 2.04 [0.51;8.16] | 544 (15.5%) | 79 (22.7%) | **2.16 [1.52;3.06]** | 45 (12.1%) | 10 (8.70%) | 0.85 [0.41;1.73] | 21 (10.0%) | 14 (17.1%) | 2.04 [0.99;4.18] | 14 (13.9%) | 2 (3.77%) | 0.27 [0.06;1.15] |
| Statin treatment |  |  |  |  |  |  |  |  |  |  |  |  |  |  |  |
| No | 1585 (89.3%) | 28 (77.8%) | Ref. | 3405 (71.7%) | 264 (55.1%) | Ref. | 267 (48.7%) | 67 (45.3%) | Ref. | 135 (46.9%) | 53 (43.1%) | Ref. | 77 (46.4%) | 52 (53.1%) | Ref. |
| Yes | 189 (10.7%) | 8 (22.2%) | **2.37 [1.08;5.21]** | 1347 (28.3%) | 215 (44.9%) | **2.00 [1.67;2.39]** | 281 (51.3%) | 81 (54.7%) | 1.08 [0.78;1.50] | 153 (53.1%) | 70 (56.9%) | 1.09 [0.77;1.56] | 89 (53.6%) | 46 (46.9%) | 0.76 [0.51;1.13] |
| Anti-hypertensive drug treatment |  |  |  |  |  |  |  |  |  |  |  |  |  |  |  |
| No | 1565 (88.2%) | 25 (69.4%) | Ref. | 3591 (75.6%) | 231 (48.2%) | Ref. | 252 (46.0%) | 53 (35.8%) | Ref. | 84 (29.2%) | 25 (20.3%) | Ref. | 27 (16.3%) | 19 (19.4%) | Ref. |
| Yes | 209 (11.8%) | 11 (30.6%) | **3.32 [1.63;6.75]** | 1161 (24.4%) | 248 (51.8%) | **3.15 [2.63;3.77]** | 296 (54.0%) | 95 (64.2%) | **1.45 [1.04;2.03]** | 204 (70.8%) | 98 (79.7%) | 1.50 [0.96;2.32] | 139 (83.7%) | 79 (80.6%) | 0.87 [0.52;1.43] |
| Antiplatelet drug treatment |  |  |  |  |  |  |  |  |  |  |  |  |  |  |  |
| No | 1716 (96.7%) | 33 (91.7%) | Ref. | 3835 (80.7%) | 302 (63.0%) | Ref. | 314 (57.3%) | 66 (44.6%) | Ref. | 150 (52.1%) | 43 (35.0%) | Ref. | 86 (51.8%) | 33 (33.7%) | Ref. |
| Yes | 58 (3.27%) | 3 (8.33%) | 2.70 [0.83;8.80] | 917 (19.3%) | 177 (37.0%) | **2.35 [1.95;2.83]** | 234 (42.7%) | 82 (55.4%) | **1.56 [1.13;2.15]** | 138 (47.9%) | 80 (65.0%) | **1.73 [1.19;2.50]** | 80 (48.2%) | 65 (66.3%) | **1.67 [1.10;2.53]** |

Data are mean [SD] for quantitative variables, and n (%) for categorical variables. ^1^HDL-cholesterol <40mg/dl in men and <50mg/dl in women. ^2^Triglycerides ≥150 mg/dl and low HDL cholesterol. ^3^ICD-10 diagnosis E10.2 to E10.4, N08.3, E36.0 or G63.2. Age groups: young (Y): <35 years; early adulthood (EA): 35 to 55/60 (men/women) years; middle adulthood (MA): 55/60 to 65 (men/women); young old (YO): 66 to 75 years and middle-to-very old (MVO): >75 years

**Supplementary Table 5**

Age and sex distribution of participants who died versus those who did not die at the end of follow-up

|  | All (n=8412) | | Men (n=4853) | | Women (n=3559) | |
| --- | --- | --- | --- | --- | --- | --- |
|  | Alive  n=7922 (93.2) | Dead  n=490 (5.8) | Alive  n=4556 (93.9) | Dead  n=297 (6.1) | Alive  n=3366 (94.6) | Dead  n=193 (5.4) |
| Age, mean (SD) | 44.2  (11.1) | 61.7  (15.7) | 43.5  (10.4) | 58.7  (14.7) | 45.1  (11.9) | 66.4  (16.1) |
| Y, n (%) | 1790 (98.9) | 20 (1.1) | 1058 (98.6) | 15 (1.4) | 732 (99.3) | 5 (0.7) |
| EA, n (%) | 5053 (96.6) | 178 (3.4) | 2837 (96.2) | 111 (3.4) | 2216 (97.1) | 67(2.9) |
| MA, n (%) | 614 (88.2) | 82 (11.8) | 461 (86.2) | 74 (13.8) | 153 (95.0) | 8 (5.0) |
| YO, n (%) | 334 (81.3) | 77 (18.7) | 158 (79.0) | 42 (21.0) | 176 (83.4) | 35 (16.6) |
| MVO, n (%) | 131 (49.6) | 133 (50.4) | 42 (43.3) | 55 (56.7) | 89 (53.3) | 78 (46.7) |

Age groups: young (Y): <35 years; early adulthood (EA): 35 to 55/60 (men/women) years; middle adulthood (MA): 55/60 to 65 (men/women); young old (YO): 66 to 75 years and middle-to-very old (MVO): >75 years

**Supplementary Table 6**

Age and sex distribution of deceased participants according to the absence/presence of a first cardiovascular event during the study period

|  | All (n=490) | | Men (n=297) | | Women (n=193) | |
| --- | --- | --- | --- | --- | --- | --- |
|  | No CVD  n=304 (62.1) | CVD  n=186 (37.9) | No CVD  n=185 (62.3) | CVD  n=112 (37.7) | No CVD  n=119 (61.7) | CVD  n=74 (38.3) |
| Age, mean (SD) | 59.5  (16.5) | 65.3  (13.7) | 56.4  (15.4) | 62.4  (12.6) | 64.3  (16.1) | 69.8  (14.2) |
| Y, n (%) | 19 (95.0) | 1 (5.0) | 14 (93.3) | 1 (6.7) | 5 (100) | 0 (0) |
| EA, n (%) | 122 (68.5) | 56 (31.6) | 76 (68.5) | 35 (31.5) | 46 (68.7) | 21 (31.3) |
| MA, n (%) | 49 (59.7) | 33 (40.3) | 45 (60.8) | 29 (39.2) | 4 (50) | 4 (50) |
| YO, n (%) | 37 (48.0) | 40 (52.0) | 18 (42.9) | 24 (57.1) | 19 (54.3) | 16 (45.7) |
| MVO, n (%) | 77 (57.9) | 56 (42.1) | 32 (58.2) | 23 (41.8) | 45 (57.7) | 33 (42.3) |

Age groups: young (Y): <35 years; early adulthood (EA): 35 to 55/60 (men/women) years; middle adulthood (MA): 55/60 to 65 (men/women); young old (YO): 66 to 75 years and middle-to-very old (MVO): >75 years
